# Myeloid Arginase 1 Insufficiency Exacerbates Amyloid-β Associated Neurodegenerative Pathways and Glial Signatures in a Mouse Model of Alzheimer’s Disease: A Targeted Transcriptome Analysis

**DOI:** 10.3389/fimmu.2021.628156

**Published:** 2021-05-11

**Authors:** Chao Ma, Jerry B. Hunt, Andrii Kovalenko, Huimin Liang, Maj-Linda B. Selenica, Michael B. Orr, Bei Zhang, John C. Gensel, David J. Feola, Marcia N. Gordon, Dave Morgan, Paula C. Bickford, Daniel C. Lee

**Affiliations:** ^1^ Department of Molecular Pharmacology and Physiology, Morsani College of Medicine, University of South Florida, Tampa, FL, United States; ^2^ Sanders-Brown Center on Aging, Department of Neuroscience, College of Medicine, University of Kentucky, Lexington, KY, United States; ^3^ Department of Pharmaceutical Sciences, College of Pharmacy, University of South Florida, Tampa, FL, United States; ^4^ Sanders-Brown Center on Aging, Department of Molecular and Cellular Biochemistry, College of Medicine, University of Kentucky, Lexington, KY, United States; ^5^ Spinal Cord and Brain Injury Research Center, Department of Physiology, College of Medicine, University of Kentucky, Lexington, KY, United States; ^6^ Center for Neurogenetics, Feil Family Brain and Mind Research Institute, Weill Cornell Medicine, Cornell University, New York, NY, United States; ^7^ Department of Pharmacy Practice and Science, College of Pharmacy, University of Kentucky, Lexington, KY, United States; ^8^ Department of Translational Neuroscience, College of Human Medicine, Michigan State University, Grand Rapids, MI, United States; ^9^ Center of Excellence for Aging and Brain Repair, Department of Neurosurgery and Brain Repair, Morsani College of Medicine, University of South Florida, Tampa, FL, United States; ^10^ Research Service, James A. Haley Veterans Affairs Hospital, Tampa, FL, United States

**Keywords:** arginine metabolism, APP Tg2576, amyloidosis, neurodegeneration, neuroinflammation, infiltrating macrophage, microglia, nCounter technology

## Abstract

Brain myeloid cells, include infiltrating macrophages and resident microglia, play an essential role in responding to and inducing neurodegenerative diseases, such as Alzheimer’s disease (AD). Genome-wide association studies (GWAS) implicate many AD casual and risk genes enriched in brain myeloid cells. Coordinated arginine metabolism through arginase 1 (*Arg1*) is critical for brain myeloid cells to perform biological functions, whereas dysregulated arginine metabolism disrupts them. Altered arginine metabolism is proposed as a new biomarker pathway for AD. We previously reported *Arg1* deficiency in myeloid biased cells using lysozyme M (LysM) promoter-driven deletion worsened amyloidosis-related neuropathology and behavioral impairment. However, it remains unclear how *Arg1* deficiency in these cells impacts the whole brain to promote amyloidosis. Herein, we aim to determine how *Arg1* deficiency driven by LysM restriction during amyloidosis affects fundamental neurodegenerative pathways at the transcriptome level. By applying several bioinformatic tools and analyses, we found that amyloid-β (Aβ) stimulated transcriptomic signatures in autophagy-related pathways and myeloid cells’ inflammatory response. At the same time, myeloid *Arg1* deficiency during amyloidosis promoted gene signatures of lipid metabolism, myelination, and migration of myeloid cells. Focusing on Aβ associated glial transcriptomic signatures, we found myeloid *Arg1* deficiency up-regulated glial gene transcripts that positively correlated with Aβ plaque burden. We also observed that Aβ preferentially activated disease-associated microglial signatures to increase phagocytic response, whereas myeloid *Arg1* deficiency selectively promoted homeostatic microglial signature that is non-phagocytic. These transcriptomic findings suggest a critical role for proper *Arg1* function during normal and pathological challenges associated with amyloidosis. Furthermore, understanding pathways that govern *Arg1* metabolism may provide new therapeutic opportunities to rebalance immune function and improve microglia/macrophage fitness.

## Introduction

Dysregulation of arginine metabolism has been recognized to impact neuropathology and neuroinflammation in Alzheimer’s disease (AD). Proper regulation of arginine metabolism through its different catabolizing enzymes (*ARG1, ARG2, NOS1, NOS2, NOS3, ADC, AGAT1, ATE1*) (1, 2) and protein sensors (*GPRC6A, SLC38A9, CASTOR1, CASTOR2, TM4SF5*) (3–6) remains critical for cellular responses to pathogenic stimuli, especially in myeloid cells. Recent metabolomics research in AD uncovered promising biomarker signatures associated with the deregulation of arginine-related pathways and polyamines in the blood and cerebral spinal fluid (CSF) (7–10). The altered metabolism of arginine and arginase expression was confirmed in AD postmortem brains (11–16). Human *ARG1* has 43 mutations linked to *ARG1* deficiency disorder, and a rare *ARG2* variant was associated with a higher risk of AD (16, 17). Increased arginine and altered *Arg1* in the brain of animal models of aging and AD were also reported, signifying a pivotal role for proper arginine metabolism in neurodegeneration (15, 18–21).

It has been reported that *Arg1* is primarily expressed in brain-infiltrating macrophages over microglia after central nervous system (CNS) injury and ischemia, and brain infiltrating macrophages relied on *Arg1* expression to exhibit a reparative role to ameliorate damages (22–26). Most recently, as a marker, *ARG1* distinguished human brain resident microglia from peripheral blood mononuclear cells (PBMCs) and cerebrospinal fluid mononuclear cells (CSF cells). The potential infiltrating CSF cells expressed the highest level of *ARG1* among all three (27). The discrepancy of *Arg1* expression between brain infiltrating macrophages and resident microglia complicates its role in myeloid cell subtypes and makes the entire brain myeloid cells (microglia/macrophages) a promising target for research and therapeutics (28–32). Albeit less studied in neurodegeneration, *Arg1* activity is essential for controlling the inflammatory response of brain myeloid cells to an extracellular stimulus like amyloid-β (Aβ). In animal models of AD, our group and several others have demonstrated that peripheral myeloid cells like monocytes could infiltrate into the brain as local tissue macrophages to clear Aβ deposits together with activated brain resident microglia (33–36). We showed that *Arg1* overexpression in the CNS decreased the inflammatory response and ameliorated tau pathology (37). Another group showed that *Arg1* positive microglia reduced Aβ plaque burden under an IL-1β dependent inflammatory condition (38). Besides, myeloid-specific knockout of *Arg1* in a retinal injury mouse model worsened neuronal loss and increased inflammatory responses (26).

Previously we sought to investigate the impact of reduced myeloid *Arg1* in the APP Tg2576 mouse model of amyloidosis (39). We found that *Arg1* insufficiency in lysozyme M (LysM) restricted cells produced more Aβ deposition, increased activated microglia, and impaired behavioral performance. Moreover, amyloidosis induced machinery of the Ragulator-Rag complex responsible for phagocytosis. However, *Arg1* deficiency blunted this response, suggesting a crucial role of arginine metabolism at the lysosome. While there is considerable evidence supporting that myeloid *Arg1* deficiency during amyloidosis exacerbates AD-typical neuropathology and behavioral impairments, the underlying mechanisms have yet to be fully clarified. We performed bulk RNA transcriptome analysis to determine the transcriptional pathway changes following myeloid *Arg1* haploinsufficiency during amyloidosis. We utilized the gene expression profiling with the nCounter^®^ mouse neuropathology panel (NanoString Technologies, Inc.) to analyze the top changed Aβ associated neurodegenerative pathways and glial signatures. In the current study, our data suggest that insufficient myeloid *Arg1* expression during amyloidosis activates transcriptomic pathways in myelination, lipid metabolism, and glial gene signatures that are primarily homeostatic and non-phagocytic. These data provide a more comprehensive transcriptional landscape of myeloid *Arg1* insufficiency during amyloidosis, which could offer new therapeutic targets that improve myeloid function to mitigate amyloid deposition.

## Materials and Methods

### Animal Breeding and Tissue Harvesting

The *APP* Tg2576 mice overexpressing human *APP KM670/671NL* Swedish mutation (40) and the non-transgenic littermates were bred at USF Health Byrd Alzheimer’s Institute at the University of South Florida. The Cre-recombinase mice (B6.129P2-*Lyz2^tm1(cre)/fo^/J; LysMcre* promoter, Stock No: 004781) and arginase 1 mice (C57BL/6*-Arg1^tm1Pmu/^*J (*Arg1^fl^*), Stock No: 008817) were purchased from the Jackson Laboratory. The *APP* Tg2576 mice (APP^+/-^), *Arg1* floxed mice (*Arg1^fl/fl^*), and *LysMcre^Tg/+^* were bred as previously described (41). Thus we established four groups of mice: *nTg/Arg1^+/+^/LysMcre^Tg/+^*, *nTg/Arg1^fl/+^/LysMcre^Tg/+^*, *APP^+/-^/Arg1^+/+^/LysMcre^Tg/+^* and *APP^+/-^/Arg1^fl/+^/LysMcre^Tg/+^*. All mice were subjected to behavioral tests at the age of 15 months, followed by euthanasia and perfusion at 16-month-old age. Immediately after perfusion, one hemisphere of the brain was placed into 4% paraformaldehyde for fixation, and the other hemisphere was dissected into different brain regions and stored at -80°C. For dissection, the cerebral cortex was peeled off the brain’s surface, taking care not to include the hippocampus, striatum, or other underlying structures. The posterior cortex, including entorhinal, temporal, parietal, and occipital areas, was collected for analysis (39).

### RNA Preparation for nCounter^®^ Gene Expression Analysis

Total RNA of the posterior cortex was extracted using AllPrep DNA/RNA/Protein Mini Kit (QIAGEN, #80004) according to the manufacturer’s protocol. The posterior cortex was selected because entorhinal to hippocampal connections are critical to memory formation and are affected early in both AD and the Tg2576 mouse model (42). This area develops a high amyloid burden in these mice, as reported before (39). All RNA samples passed QC with high RNA Integrity Number (RIN) measured using TapeStation RNA Screen Tape^®^ (Agilent Technologies, Inc., Molecular Genomics Core, Moffitt Cancer Center, Tampa, FL, US). For NanoString nCounter^®^ analysis, we pooled an equal mass of RNA from two mice matched for genotype, gender, and age, so one pooled RNA sample represents two mice. Thus we created the following four groups of RNA samples: *nTg/Arg1^+/+^/LysMcre^Tg/+^* (4 males/2 females, average RIN = 8.4), *nTg/Arg1^fl/+^/LysMcre^Tg/+^* (2 males/4 females, average RIN = 8.8), *APP^+/-^/Arg1^+/+^/LysMcre^Tg/+^* (4 males/2 females, average RIN = 8.7), and *APP^+/-^/Arg1^fl/+^/LysMcre^Tg/+^* (4 males/2 females, average RIN = 8.7). Each group contained three samples representing six mice. The nCounter^®^ mouse neuropathology panel (v1.0, XT-CSO-MNROP1-12, 12 Reactions, NanoString Technologies, Inc.) was purchased for analyzing 12 mouse brain samples. The panel plate loaded with RNA samples was analyzed by the nCounter^®^ Analysis System (nCounter^®^ SPRINT Profiler at the Molecular Genomics Core, Moffitt Cancer Center, Tampa, FL, US) according to the manufacturer’s procedures for hybridization, detection, and scanning.

### Bioinformatic Analyses

#### The nSolver Analysis

The NanoString nCounter data analysis was performed using the nSolver™ Analysis Software (v4.0, NanoString Technologies, Inc.) according to the user manuals (43, 44). The nSolver was used for analyzing the mouse neuropathology panel by loading with an RLF file (NS_Mm_NeuroPath_v1.0). We performed the basic features of the nSolver analysis to generate the heat maps of differentially expressed gene transcripts (DEGs) with agglomerative clustering based on the normalized data. We also used it to export all normalized data. We also performed the advanced nSolver analysis based on the downloaded and installed online R package (nCounter_Advanced_Analysis_2.0.134) and the probe annotation file provided by NanoString (NS_Mm_NeuroPath_v1.0_ProbeAnnotations). We modified this probe annotation file as the following description. First, we annotated two autophagy genes (*Atg5, Becn1*) to the autophagy pathway. Second, we referred to the so far most comprehensive GWAS publications in AD and annotated 15 gene transcripts into a new pathway named “AD Causal Risk Pathway” (*Cntnap2, Cd33, Psen2, Mapt, Psmb9, App, Psen1, Apoe, Trem2, Adam10, Psmb8, Spi1, Sorl1, Clu, C4a*) (45–49). All RNA samples passed system QC parameters on imaging (fields of view > 75), binding density (0.1-2.25), and positive control linearity (R^2^ > 0.95). All RNA data passed the positive control limit of detection QC (0.5fM > 2*standard deviations above the arithmetic mean of the negative controls). The following basic threshold criteria were applied. A threshold count value of 10 was calculated as the gene transcript expression background (arithmetic mean of negative controls + 2*standard deviation). An observation frequency of 0.5 was applied for the background, indicating gene transcripts with counts lower than 10 were omitted if it happened to more than 50% of the samples. Therefore, 648 genes were above the background, and 122 genes were removed for falling below the background too frequently. A total of 6 house-keeping genes (*Aars, Ccdc127, Cnot10, Csnk2a2, Lars, Mto1*) were selected for data normalization by the geNorm algorithm (50). The *p*-values were adjusted for multiple testing by the false discovery rate (FDR) method of Benjamini and Hochberg correction (51). The matrix remodeling pathway was dropped from pathway scoring analysis due to less than five detected gene transcripts. In gene set analysis, a directed global significance score at ±1.3 was set as the cut-off value to reflect the top changed pathways further. Pathway scoring analysis and gene set analysis could independently assess a transcriptomic pathway’s change due to different algorithms.

#### Selection of Genes for nSolver Cell-type Profiling Analysis

In cell-type profiling analysis, we performed advanced nSolver analysis to select cell-type-specific marker gene transcripts to characterize the major central nervous system cells (see **Figure 4A**). We identified astrocytes (52–54), endothelial cells (53), microglia/macrophages (53–55), neurotransmitter-secreting neurons (53), oligodendrocytes (53), and mature neurons (53, 54). To clarify, the cell type scores can only be interpreted as relative cell abundance values rather than quantitative cell abundance in a group. Due to the counting and capturing efficiencies of individual cell-type-specific gene transcripts, one cell type score can be compared to the same cell type among different groups, but it cannot compare different cell types within the same group.

### Selection of Focus Genes Indicative of Amyloid-β Associated Glial Transcriptomic Signatures for nSolver Analyses

Data from published transcriptomic studies using mouse models of amyloidosis were consulted to identify seven amyloid-β associated glial transcriptomic signatures. We cross-referenced these signature top prioritized genes with the NanoString mouse neuropathology panel (**Figure 5A**). We selected 17 out of 57 gene transcripts that overlap with Plaque Induced Genes (PIGs; purple WGCNA module genes in reference Table S3) and 30 out of 165 that overlap with Plaque Correlated Oligodendrocyte Genes (OLIGs; red WGCNA module genes in reference Table S3) (56). We selected 15 out of the top 117 that overlap with Disease-associated Microglia (DAM; sorted by *p*-values, -log10 (*p*-value) ≥ 21, up-regulated genes of “MG3/MG1” in reference Table S2 and Figure 6) and 9 out of total 36 that overlap with Homeostatic Microglia (HM; sorted by *p*-values, -log10 (*p*-value) ≥ 7, down-regulated genes of “MG3/MG1” in reference Table S1 and Figure 6) (57). We selected 20 out of total 114 that overlap with Microglial Neurodegenerative Phenotype (MGnD; sorted by fold-change, log2 (FC) ≥ 0.01, up-regulated genes of “APP-PS1 10mo/WT 10mo” in reference Table S1) and 25 out of top 169 that overlap with Tolerogenic Microglia (M0; sorted by fold-change, log2 (FC) ≤ -0.43, down-regulated genes of “APP-PS1 10mo/WT 10mo” in reference Table S1) (58). We selected 15 out of the top 121 that overlap with Disease-associated Astrocytes (DAA; sorted by *p*-values, -log10 (*p*-value) ≥ 21, up-regulated genes of “Cluster 4/Cluster 1” in reference Table S2) (59).

#### IPA Analysis

Ingenuity^®^ Pathway Analysis (IPA^®^, 2000-2020, QIAGEN) was applied for functional enrichment analysis and causal analysis using the differentially expressed gene transcripts (DEGs, *p* < 0.05), as previously described (60, 61). IPA core analysis was performed to determine the top canonical pathways, predict upstream regulators, diseases/disorders, and biological functions based on two statistical measures (*p*-value and *z*-score). The *p*-value calculated the statistical significance of the overlap between the current dataset and the publicly available databases by a Fisher’s exact test. The *z*-score implicates how likely the predicted state is activated/increased (z ≥ 2) or inhibited/decreased (z ≤ -2) based on a comparison model. The *p*-values less than 0.05 were enforced for all analyses in IPA. For analysis in canonical pathways, pathways were sorted based on *p*-values, and the top five most significant pathways were listed. For upstream analysis, upstream regulators were sorted by *z*-score to focus on predicted activation (z ≥ 2) or inhibition (z ≤ -2). A gene interaction network was plotted, including the predicted upstream regulators and associated target gene transcripts. IPA regulator effect analysis suggested potential mechanisms. Diseases or functions were sorted by *z*-score to show predicted increased (z ≥ 2) or decreased (z ≤ -2) risk. To reduce data redundancy, overlapped items (pathways/diseases/functions) were removed if they shared the same target gene transcripts and linked to unrelated peripheral organs/cancer/tumors.

#### STRING Analysis

STRING (Version 11.0), part of the ELIXIR Core Data Resources, is known for its predicted protein-protein interactions but also analyzes mRNA gene transcript in the current version (62). STRING was applied for network analysis and functional enrichment analysis using differentially expressed gene transcripts (DEGs, *p* < 0.05) with log2 fold change (FC) values. The Normal Gene Set Analysis was performed in STRING based on the mouse organism (Mus musculus, NCBI taxonomy Id: 10090). In our STRING network, nodes represent gene transcripts, and edges represent the expressed protein-protein association. The halo color of the nodes was based on the rank of DEG log2 FC. For setting STRING analysis, the network of edges was built on evidence by interaction lines (textmining, experiments, databases, co−expression) with medium confidence (0.4). Disconnected nodes were hidden in the network. STRING network with protein-protein interaction (PPI) enrichment *p*-value less than 0.05 indicates statistical significance, suggesting the expressed genes were biologically connected as a group. Functional enrichment analysis by Reactome Pathways was sorted based on an FDR *q*-value less than 0.01 and a minimum observed gene count of 5.

### Statistical Analysis

All statistical analyses were performed by SPSS (version 25.0, IBM Corp., Armonk, NY, USA). GraphPad Prism (version 8.4.3, GraphPad Software, San Diego, CA, USA) was used for generating graphs. In all cases, two-way ANOVA (2*2 factorial) was chosen to measure any main genotype effect in *APP* transgene (*APP^+/-^/LysMcre^Tg/+^* vs. *nTg/LysMcre^Tg/+^*) or *Arg1* haploinsufficiency (*Arg1^fl/+^/LysMcre^Tg/+^* vs. *Arg1^+/+^/LysMcre^Tg/+^*), and the interaction of the two genotypes. Two-way ANOVA was followed by a pair-wise comparison test using Fisher’s PLSD to investigate further the two focused comparisons (*APP^+/-^/Arg1^+/+^/LysMcre^Tg/+^* vs. *nTg/Arg1^+/+^/LysMcre^Tg/+^*; *APP^+/-^/Arg1^fl/+^/LysMcre^Tg/+^* vs. *APP^+/-^/Arg1^+/+^/LysMcre^Tg/+^*).

## Results

### Experimental Design and Workflow of Gene Expression Profiling of Mouse Brains With *APP* Transgene and *Arg1* Haploinsufficiency

**Figure 1:**
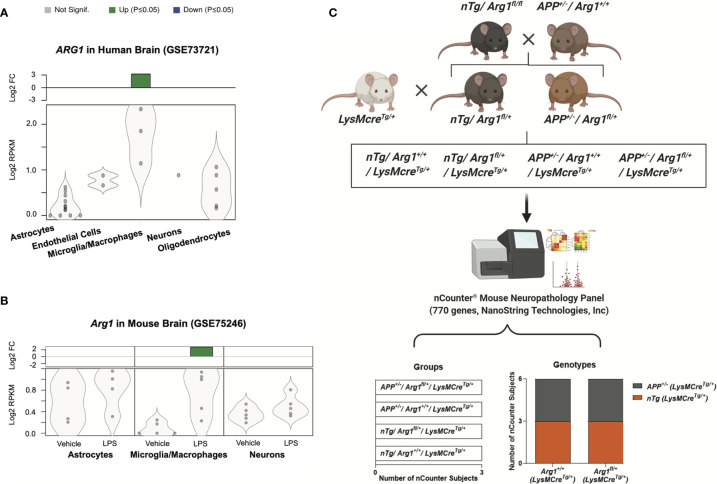
Arginase 1 gene expression in central nervous system cell types and the experimental workflow for mouse breeding and gene expression analysis. A search for RNA sequencing data sets enriched for purified central nervous system cell types from human and mouse brains identified two studies on arginase 1 expression. Datasets were obtained from the National Center for Biotechnology Information (NCBI) Gene Expression Omnibus (GEO). Gene transcripts were measured by reads per kilobase per million total reads (log2 RPKM) and presented as violin plots with dots representing samples. The green bars at the top indicate significant up-regulation (*p* ≤ 0.05) with the fold change (log2 FC) relative to the control. **(A)** The human *ARG1* gene transcript in purified cell types of normal adult human brains (GSE73721; Astrocytes, n=12; Endothelial Cells, n=2; Microglia/Macrophages, n=3; Neurons, n=1; Oligodendrocytes, n=5). **(B)** The *Arg1* gene transcript in purified cell types of normal adult mouse brains injected with LPS (GSE75246; Astrocytes, Microglia/Macrophages, Neurons; Vehicle, n=5; LPS, n=5). **(C)** The schematic representation of the experimental workflow. The *APP* Tg2576 *(APP^+/-^/Arg1^+/+^)* mice were bred with non-transgenic *Arg1 LoxP* (*nTg/Arg1^fl/fl^*) mice to generate *nTg*/*Arg1^fl/+^* mice and *APP^+/-^/Arg1^fl/+^* mice. Both were then bred with *LysMcre^Tg/+^* mice to generate a total of four groups for the study. We harvested mouse brain RNA and performed gene expression profiling using the nCounter^®^ mouse neuropathology panel (770 genes, NanoString Technologies, Inc.). Bioinformatic analyses were performed to investigate the four groups of mice (*nTg/Arg1^+/+^/LysMcre^Tg/+^*, *nTg/Arg1^fl/+^/LysMcre^Tg/+^*, *APP^+/-^/Arg1^+/+^/LysMcre^Tg/+^*, *APP^+/-^/Arg1^fl/+^/LysMcre^Tg/+^*, n=3 samples per group representing 6 mice), which cover two genotypes categorized into the *APP* transgene genotype (*APP^+/-^* vs *nTg*) and the *Arg1* haploinsufficiency genotype (*Arg1^fl/+^* vs *Arg1^+/+^*).

To investigate the gene expression of arginase 1 in CNS cell types, we performed data-mining on 35 publicly available RNA sequencing (RNA-seq) studies using purified cell types from human and mouse brains (55, 63). We found *ARG1* gene transcript was particularly up-regulated in microglia/macrophages (log2 FC = 3.228, adjusted *p* = 0.0006) but not in other CNS cell types in normal adult human brains (GSE73721) (**Figure 1A**) (64). We also found *Arg1* gene transcript was only up-regulated in microglia/macrophages (CD11b+, log2 FC = 2.777, adjusted *p* = 0.0067) in response to LPS stimulation in normal adult mouse brains, but not in astrocytes and neurons (GSE75246) (**Figure 1B**) (65). Thus, we uncovered the arginase 1 transcript in these two RNA-seq datasets and found arginase 1 was mainly enriched and active in brain microglia/macrophages, aligning with our previous findings (39, 66–68). Therefore, we sought to repress *Arg1* in myeloid cells to assess the impact of *Arg1* deficiency in the mouse brain during the amyloidosis challenge.

To target myeloid cells, we chose to use mice with the lysozyme M Cre-recombinase (LysMcre) knock-in/knockout allele, which expresses mainly in myelomonocytic cells of the myeloid lineage (monocytes, macrophages, microglia, and granulocytes) (69–72). We presented a schematic design on cross-breeding mice of *Arg1 LoxP* (non-transgenic, *nTg/Arg1^fl/fl^*) with APP Tg2576 mice (*APP^+/-^*/*Arg1^+/+^)* to produce *nTg/Arg1^fl/+^* and *APP^+/-^/Arg1^fl/+^* mice, both of which were further bred with LysMcre mice (*LysMcre^Tg/+^*) to generate four groups of mice for the study (**Figure 1C**) (72).

We extracted total RNA from the posterior cortex and pooled two samples segregated by genotype, gender, and age. Then, we performed digital RNA profiling using the nCounter^®^ mouse neuropathology panel with coverage of 770 genes to investigate the transcriptomic pathway alterations in neurodegenerative diseases like AD (73–76) and common microglial gene expression signatures (77, 78). We utilized the nCounter^®^ analysis system to directly count and quantify the abundance of RNA molecules with high reproducible results (79, 80). This unique RNA measuring technique permits a higher sensitivity than traditional real-time quantitative reverse transcription PCR (qRT-PCR) (81, 82) and microarrays (83, 84) without cDNA reverse transcription and amplification, while also allowing to procure data much faster than RNA-seq (85) and single-cell RNA-seq (scRNA-seq) (86) without cDNA library preparation (87). Finally, we performed strategic analyses in bioinformatics and statistics based on the four experimental groups in the order of *nTg/Arg1* sufficient mice (*nTg/Arg1^+/+^/LysMcre^Tg/+^*), *nTg/Arg1* insufficient mice (*nTg/Arg1^fl/+^/LysMcre^Tg/+^*), *APP*/*Arg1* sufficient mice (*APP^+/-^/Arg1^+/+^/LysMcre^Tg/+^*), and *APP*/*Arg1* insufficient mice (*APP^+/-^/Arg1^fl/+^/LysMcre^Tg/+^*). Four groups were also branched into two covariates of genotype: *APP* transgene genotype comparing *APP* mice (*APP^+/-^*) to *nTg* mice (*nTg*) and *Arg1* haploinsufficiency genotype comparing *Arg1* insufficient mice (*Arg1*_fl/+_) to *Arg1* sufficient mice (*Arg1*_+/+_; **Tables S1–S4**) (**Figure 1C**).

The heat map of all normalized data displayed mouse clustering under two genotypes with 648 gene transcripts measured above the background expression threshold (**Figure S1A**). All samples passed QC metrics and were automatically segregated by the two genotypes (**Figure S1A**). Notably, samples were correctly clustered by the *APP* transgene genotype. Principal component analysis (PCA) calculated the first four principal components (PCs) of all data with PC1 (20%), PC2 (11%), PC3 (10%), and PC4 (9%) plotting to each other against the four groups (**Figure S1B**). Accounting for the highest percentage of genotype variance, the PC1 identified modest but clear sample separation due to the *APP* transgene genotype, but to a lesser extent for the *Arg1* haploinsufficiency genotype (**Figure S1B**). It suggests that the overexpression of human *APP* transgene dominates the overall differential expression of genes, whereas the myeloid *Arg1* haploinsufficiency becomes a secondary influencing factor.

### Pathway Scoring Analysis of Fundamental Neurodegeneration Pathways in Mouse Brains With *APP* Transgene and *Arg1* Haploinsufficiency

**Figure 2:**
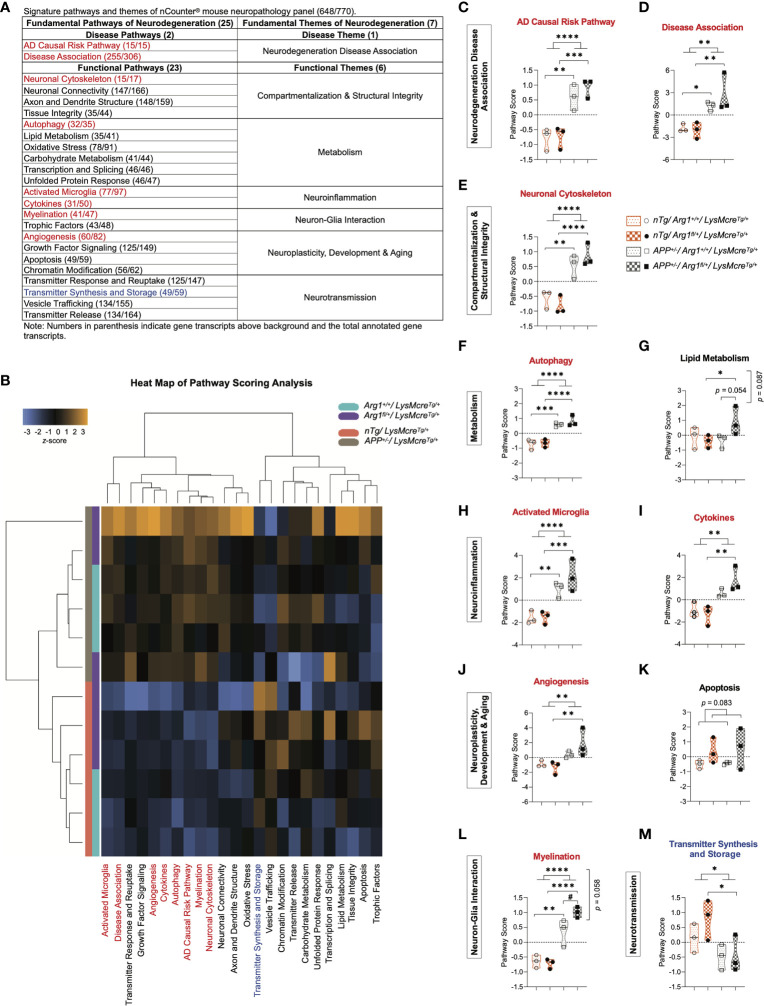
Pathway scoring analysis of fundamental neurodegeneration pathways. **(A)** A table of signature pathways and themes of nCounter^®^ mouse neuropathology panel. A total of 25 fundamental signature pathways are mapped into seven themes covering one disease theme and six functional themes. **(B)** A heat map of pathway scoring analysis provides an overview of pathway distribution and clustering across all samples. Score color in orange or blue indicates up or down-regulation of each pathway in each sample, respectively. All scores are presented on the same scale *via* a *z*-transformation. **(C, D)** Scores for pathways of AD causal risk pathway **(C)** and disease association **(D)** under the theme of neurodegeneration disease association. **(E)** Score for the pathway of neuronal cytoskeleton under the theme of compartmentalization & structural integrity. **(F, G)** Scores for pathways of autophagy **(F)** and lipid metabolism **(G)** under the theme of metabolism. **(H, I)** Scores for pathways of activated microglia **(H)** and cytokines **(I)** under the theme of neuroinflammation. **(J, K)** Scores for pathways of angiogenesis **(J)** and apoptosis **(K)** under the theme of neuroplasticity, development & aging. **(L)** Score for the pathway of myelination under the theme of neuron-glia interaction. **(M)** Score for the pathway of transmitter synthesis and storage under the theme of neurotransmission. All samples are displayed in violin plots with the median denoted as a line. Within each pathway, score values from four groups have been centered to a mean of zero. Pathways that show statistically significant main *APP* transgene genotype effects are highlighted in red or blue to indicate up or down-regulation, respectively. n=3 samples per group representing 6 mice. The asterisk sign (*) indicates the main effect of *APP* transgene genotype and its pair-wise comparisons. The number sign (#) indicates the main effect of the *Arg1* haploinsufficiency genotype and its pair-wise comparisons. Interaction of the two genotypes is indicated vertically by the *p* values. *^/#^ *p <* 0.05; **p < 0.01; ***p < 0.001; ****p < 0.0001. Two-way ANOVA of 2x2 factorial analysis followed by pairwise comparisons using Fisher’s PLSD.

All genes were annotated in 25 gene signature pathways of neurodegeneration (two disease pathways and 23 functional pathways) that were categorized into seven fundamental themes (one disease theme and six functional themes) (**Figure 2A**). To analyze each gene signature pathway, pathway scoring analysis (PSA) was performed to create a score for each group using the first principal component dataset of the pathway’s gene set (44, 88). The sum of all four groups equilibrated to zero value in each pathway, while their scores indicated up or down-regulation relative to each other. Collectively, pathway scores were visualized in a heat map to represent all pathways’ clustering across samples under two genotypes. We observed clustering of the *APP* transgene genotype between *APP* mice (*APP^+/-^/LysMcre^Tg/+^*) and *nTg* mice (*nTg/LysMcre^Tg/+^*), and clear sub-clustering of *Arg1* insufficient mice (*Arg1_fl/+_/LysMcre_Tg/+_*) and *Arg1* sufficient mice (*Arg1_+/+_/LysMcre_Tg/+_*) except for one sample (**Figure 2B**).

Then we statistically analyzed the 25 gene signature pathway scores and described them here. Compared to *nTg* mice, we found that *APP* mice up-regulated eight pathways and down-regulated one pathway due to the main genotype effect in *APP* transgene. The eight up-regulated pathways were AD causal risk pathway (*p <* 0.0001, **Figure 2C**), disease association (*p =* 0.002, **Figure 2D**), neuronal cytoskeleton (*p <* 0.0001, **Figure 2E**), autophagy (*p <* 0.0001, **Figure 2F**), activated microglia (*p <* 0.0001, **Figure 2H**), cytokines (*p =* 0.002, **Figure 2I**), angiogenesis (*p =* 0.010, **Figure 2J**), and myelination (*p <* 0.0001, **Figure 2L**). The one down-regulated pathway was transmitter synthesis and storage (*p =* 0.021; **Figure 2M**). On the other side, we did not find any main genotype effect in *Arg1* haploinsufficiency except for an increasing trend in apoptosis (*p =* 0.083, **Figure 2K**). However, we observed increased myelination (*p =* 0.023, **Figure 2L**) and an increasing trend in lipid metabolism (*p =* 0.054, **Figure 2G**) with the pairwise comparison of *APP/Arg1* insufficient mice to *APP/Arg1* sufficient mice. We also found an increased trend for two genotypes’ interaction in myelination (*p =* 0.058, **Figure 2L**) and lipid metabolism (*p =* 0.087, **Figure 2G**). In addition, the heat map of the correlation matrix of all gene signature pathways clearly separated pathways of up-regulation and down-regulation into the opposite direction (**Figure S1C**). To summarize, the *APP* transgene exerted stronger effects on gene expression than did *Arg1* haploinsufficiency, mostly by increasing transcriptomic pathways associated with the risk of neurodegeneration diseases (AD causal risk/disease association) and neuroinflammation (activated microglia/cytokines).

### Gene Set Analysis for Measuring Differential Expression of Neurodegeneration Pathways in Mouse Brains With *APP* Transgene and *Arg1* Haploinsufficiency

**Figure 3:**
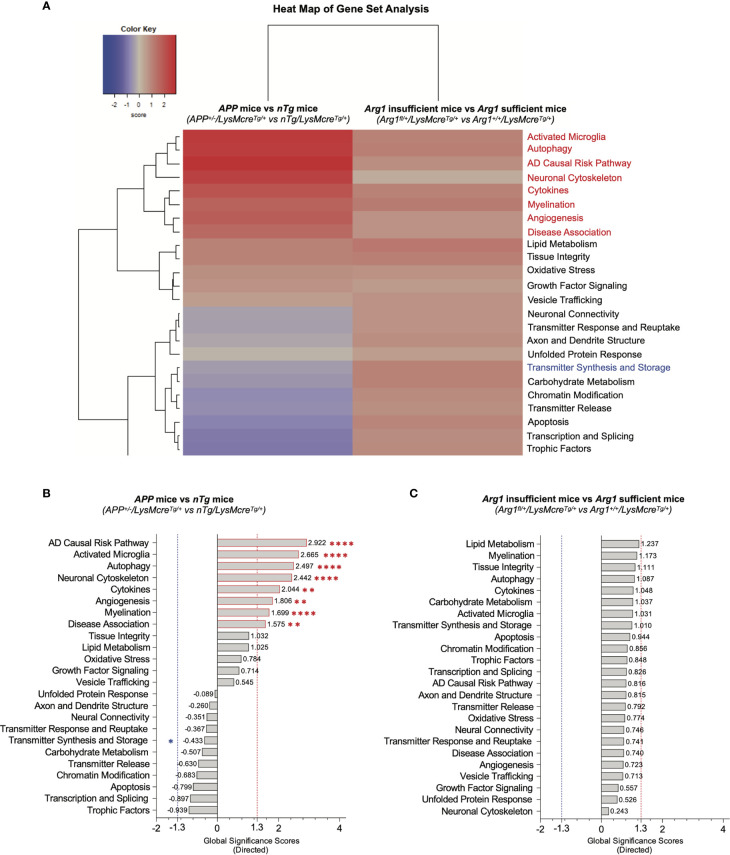
Gene set analysis for measuring overall pathway differential expression. **(A)** A heat map of directed global significance scores (GSS) against covariate of *APP* transgene genotype (*APP^+/-^/LysMcre^Tg/+^* vs *nTg/LysMcre^Tg/+^*) and *Arg1* haploinsufficiency genotype (*Arg1^fl/+^/LysMcre^Tg/+^* vs *Arg1^+/+^/LysMcre^Tg/+^*). All gene sets are presented on the same scale *via* a *z*-transformation. Gene sets that show statistically significant main *APP* transgene genotype effects from pathway scoring analysis are highlighted in red or blue to indicate up or down-regulation, respectively. **(B)** A bar graph of *APP* transgene genotype ranks all signature pathways with directed GSS values. **(C)** A bar graph of *Arg1* haploinsufficiency genotype ranks all signature pathways with directed GSS values. The up or down regulated pathway is indicated by positive or negative GSS values, respectively. The red or blue dashed line highlights the up or down regulated pathways based on the cut-off criterion (absolute GSS value at 1.3). Also, the statistically significant pathways of main *APP* transgene genotype effect identified in pathway scoring analysis are additionally annotated with red or blue asterisk sign (*). *p < 0.05; **p < 0.01; ****p < 0.0001.

To assess changes of signature pathways in *APP* transgene and *Arg1* haploinsufficiency genotypes, we performed gene set analysis (GSA) to measure the cumulative evidence for the differential expression of a pathway’s gene set. GSA calculates a directed global significance score (GSS) for each pathway based on the square root of the mean (signed) squared t-statistic of the gene set’s genes (44). Positive or negative GSS values indicate a pathway is overall up or down-regulated, respectively. We also adopted a GSS threshold value of ± 1.3 to indicate significance. The GSA heat map clearly segregated pathways of up-regulation and down-regulation (**Figure 3A**).

Comparing *APP* mice (*APP^+/-^/LysMcre^Tg/+^*) to *nTg* mice (*nTg/LysMcre^Tg/+^*), the top eight up-regulated transcriptomic pathways were AD causal risk pathway (GSS = 2.922), activated microglia (GSS = 2.665), autophagy (GSS = 2.497), neuronal cytoskeleton (GSS = 2.442), cytokines (GSS = 2.044), angiogenesis (GSS = 1.806), myelination (GSS = 1.699), and disease association (GSS = 1.575) (**Figure 3B**). Interestingly, these eight pathways were also increased in PSA due to the main genotype effect in *APP* transgene (*p* < 0.05, **Figures 2C–F, H–J, L**). However, we did not find changed pathways that met the cut-off GSS significance values when comparing *Arg1* insufficient mice (*Arg1_fl/+_/LysMcre_Tg/+_*) to *Arg1* sufficient mice (*Arg1_+/+_/LysMcre_Tg/+_*) (**Figure 3C**). In summary, two different pathway scoring algorithms (PSA and GSA) identified the overlapped up-regulated transcriptomic pathways enriched in neurodegeneration disease association (AD causal risk pathway/disease association), neuroinflammation (activated microglia/cytokines), and others (autophagy/neuronal cytoskeleton/angiogenesis/myelination), caused by the human *APP* transgene.

### Cell-type Profiling Analysis of Central Nervous System Reveals *APP* Transgene Activates Microglia/Macrophages and Myeloid *Arg1* Deficiency During Amyloidosis Promotes Oligodendrocytes

**Figure 4:**
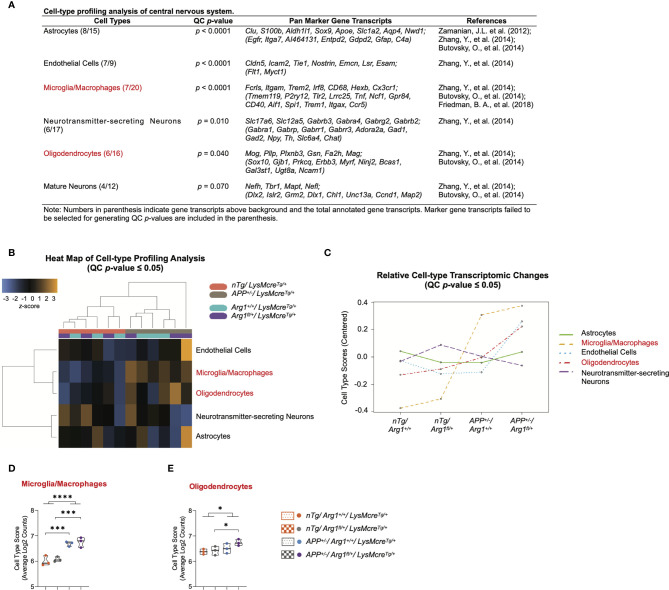
Cell-type profiling analysis of the central nervous system. **(A)** A table lists central nervous system cell types, QC *p*-value, pan marker gene transcripts, and references. **(B)** Heat map of cell-type profiling analysis provides an overview of cell-type distribution and clustering across all samples within two covariates of genotype. The *z*-score color in orange or blue indicates a high or low abundance of cell-type-specific gene transcripts, respectively. All scores are presented on the same scale *via* a *z*-transformation. **(C)** A line plot of relative cell-type transcriptomic changes in selected marker gene transcripts’ abundance across groups. Each cell type score was centered to zero value. **(D, E)** Cell type scores of microglia/macrophages **(D)** and oligodendrocytes **(E)** are displayed in a violin plot against four groups. All samples are displayed, and the median is denoted with a line. n=3 samples per group representing 6 mice. Cell types highlighted in red indicates main *APP* transgene genotype effect in cell type scores. The asterisk sign (*) indicates the main effect of *APP* transgene genotype and its pair-wise comparisons. *p < 0.05; ***p < 0.001; ****p < 0.0001. Two-way ANOVA of 2x2 factorial analysis followed by pairwise comparisons using Fisher’s PLSD.

We performed a cell-type profiling analysis (CPA) on CNS cells using their cell-type-specific gene transcripts to explore which cell types changed across four groups due to the two covariates of genotype. We first applied a QC *p*-value to investigate the validity of measuring each cell type. Statistically significant cell types (QC *p* ≤ 0.05) indicate the selected marker gene transcripts exhibit a significant cell-type-specific correlation than randomly selected genes with a similar size. This stringent algorithm requires selecting cell-type-specific genes that are consistently expressed above background while keeping a correlation expression slope close to 1 (44, 89). Therefore, we confidently characterized cell types of astrocytes (QC *p <* 0.0001), endothelial cells (QC *p <* 0.0001), microglia/macrophages (QC *p <* 0.0001), neurotransmitter-secreting neurons (QC *p =* 0.010), oligodendrocytes (QC *p =* 0.040), but not the mature neurons (QC *p =* 0.060) (**Figure 4A**). The CPA heat map presented the clustering of cell types (QC *p* ≤ 0.05) corresponding to genotypes of *APP* transgene and *Arg1* haploinsufficiency (**Figure 4B**). The CPA line plot showed the relative cell-type transcriptomic changes in selected marker gene transcripts’ abundance across groups (**Figure 4C**).

Furthermore, the average log2 counts of the selected marker gene transcripts were calculated as the cell type scores. Comparing *APP/Arg1* sufficient mice with *nTg/Arg1* sufficient mice, we observed increased cell type score only in microglia/macrophages (*Fcrls, Itgam, Trem2, Irf8, CD68, Hexb, Cx3cr1*; *p =* 0.001, **Figure 4D**), but not other CNS cell types including oligodendrocytes (*Mog, Pllp, Plxnb3, Gsn, Fa2h, Mag*; *p =* 0.351, **Figure 4E**). Surprisingly, *APP/Arg1* insufficient mice showed increased cell type score over the *nTg/Arg1* insufficient mice in both microglia/macrophages (*p =* 0.001, **Figure 4D**) and oligodendrocytes (*p =* 0.038, **Figure 4E**). Therefore, both microglia/macrophages (*p <* 0.0001, **Figure 4D**) and oligodendrocytes (*p =* 0.04, **Figure 4E**) suggested main effects in *APP* transgene genotype. Collectively, the CPA data strongly suggest that *APP* transgene activates microglia/macrophages, and myeloid *Arg1* deficiency during amyloidosis promotes oligodendrocytes, by which CNS cell types presumably drive the regulation of neurodegeneration.

### Myeloid *Arg1* Deficiency During Amyloidosis Enhances Amyloid-β Associated Glial Transcriptomic Signatures Biased for Promoting Homeostatic Microglial Genes

**Figure 5:**
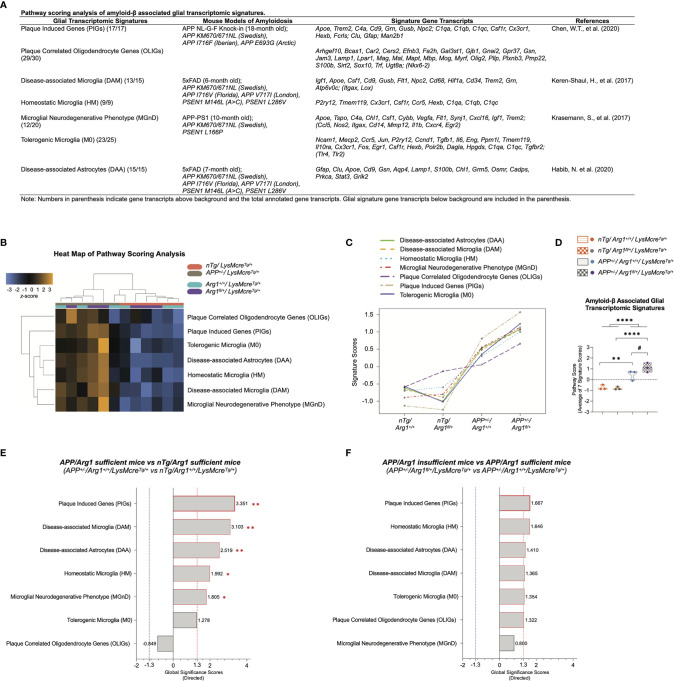
Pathway scoring analysis of amyloid-β associated glial transcriptomic signatures. **(A)** A table lists seven key glial transcriptomic signatures attributed to amyloid-β stimulus described in recent milestone publications using different mouse models of amyloidosis. **(B)** A heat map of pathway scoring analysis provides an overview of the glial signature distribution and sample clustering. The *z*-score color in orange or blue indicates up or down-regulation of glial signature by each sample. All scores are presented on the same scale *via* a *z*-transformation. **(C)** A line plot of seven glial signatures’ scores shows the transcriptomic changes across groups. Signature cores have been centered to a value of zero. **(D)** The score of amyloid-β associated glial transcriptomic signatures is summarized as the average of seven glial signatures’ scores. **(E, F)** Gene set analysis is presented in bar graphs using directed global significance scores (GSS) by comparing *APP^+/-^/Arg1^+/+^/LysMcre^Tg/+^* to *nTg/Arg1^+/+^/LysMcre^Tg/+^* **(E)** and *APP^+/-^/Arg1^fl/+^/LysMcre^Tg/+^* to *APP^+/-^/Arg1^+/+^/LysMcre^Tg/+^* **(F)**. The red or blue dashed line highlights the top up or down regulated signatures that meet the cut-off criterion (absolute GSS value at 1.3). Also, the statistically significant signatures from pathway scoring analysis are additionally annotated with red asterisk sign (*). n=3 samples per group representing 6 mice. The asterisk sign (*) indicates the main effect of *APP* transgene genotype and its pair-wise comparisons. The number sign (#) indicates the main effect of the *Arg1* haploinsufficiency genotype and its pair-wise comparisons. *^/#^ *p* < 0.05, **p < 0.01; ****p < 0.0001. Two-way ANOVA of 2x2 factorial analysis followed by pairwise comparisons using Fisher’s PLSD.

Recent progress in studying glia (microglia/astrocytes/oligodendrocytes) using scRNA-seq techniques has established specific transcriptomic signature identities and pathophysiological roles in mouse models of neurodegenerative diseases, including AD (90–92). Therefore, disease-associated microglia (DAM) (57), microglial neurodegenerative phenotype (MGnD) (58), and disease-associated astrocytes (DAA) (59) were all considered disease-associated phagocytic glial cells commonly induced in responding to Aβ plaques. Meanwhile, they suppressed non-phagocytic glial cells like homeostatic microglia (HM) (57) and tolerogenic microglia (M0) (58). Most recently, novel signatures of plaque induced genes (PIGs) and plaque correlated oligodendrocyte genes (OLIGs) were found positively correlated with Aβ plaque burden (56). The PIGs were mainly expressed by microglia and astrocytes that closely interacted with Aβ plaques, while oligodendrocytes primarily expressed the OLIGs in regulating myelination. Interestingly, the PIGs shares 41% of genes with DAM/MGnD signatures (*Apoe, Trem2, C4a, Cd9, Grn, Gusb, Npc2*), 41% with HM/M0 signatures (*C1qa, C1qb, C1qc, Csf1r, Cx3cr1, Hexb, Fcrls*), 12% with DAA signature (*Clu, Gfap*), and 6% of others (*Man2b1*), strongly suggesting Aβ associated glial transcriptomic signatures are a mix of glial genes covering disease-associated state and homeostatic state (**Figure 5A**).

We have shown that brain myeloid cells (microglia/macrophages) express the highest arginase 1 among CNS cell types (**Figures 1A, B**), and they were also activated in *APP* mice (**Figure 4D**). This would suggest that *Arg1* deficiency in myeloid cells may affect their normal immune response and reparative role upon various stimuli. It is unknown if myeloid *Arg1* deficiency impacts the glial signatures during the challenge of the Aβ stimulus. To address this question, we systematically matched our data with those above seven major published Aβ associated glial gene signatures solely characterized in mouse models of amyloidosis (**Figure 5A**).

We first performed PSA and displayed it in a heat map, indicating the significant role of *APP* transgene in segregating the *APP* mice from the *nTg* mice with extensive gene signature overexpression (**Figure 5B**). We also presented a line plot showing that the *APP* mice had an increased signature score than the *nTg* mice, whereas the *APP/Arg1* insufficient mice kept the highest score in all glial signatures than any other groups (**Figure 5C**). Collectively, we collated the seven signatures and merged them as amyloid-β associated glial transcriptomic signatures. We observed an increased signature score in the main genotype effect of *APP* transgene (*p <* 0.0001) and its pairwise comparison between *APP/Arg1* sufficient mice and *nTg/Arg1* sufficient mice (*p =* 0.003). Most importantly, the *APP/Arg1* insufficient mice had an increased score than the *APP/Arg1* sufficient mice (*p =* 0.049). (**Figure 5D**).

Combining PSA with GSA, we found gene signatures of PIGs (*p* = 0.002, GSS = 3.351), DAM (*p* = 0.004, GSS = 3.103), DAA (*p* = 0.005, GSS = 2.519), HM (*p* = 0.048, GSS = 1.992), and MGnD (*p* = 0.024, GSS = 1.805) were up-regulated comparing *APP/Arg1* sufficient mice to *nTg/Arg1* sufficient mice (**Figure 5E**). Although no significance in PSA, we discovered gene signatures of PIGs (GSS = 1.667), HM (GSS = 1.646), DAA (GSS = 1.410), DAM (GSS = 1.365), M0 (GSS = 1.354), and OLIGs (GSS = 1.322) were up-regulated in GSA comparing *APP/Arg1* insufficient mice to *APP/Arg1* sufficient mice (**Figure 5F**). Notably, myeloid *Arg1* deficiency in *APP* mice further activated the PIGs as its top changed signature while promoting HM as the second (**Figure 5F**). To summarize, these data suggest that myeloid *Arg1* deficiency enhances Aβ associated glial transcriptomic signatures leaning towards homeostatic and non-phagocytic directionality.

### Human *APP* Transgene Elevates Gene Signatures Associated With Autophagy, Activated Microglia and Inflammatory Response of Myeloid Cells in Mouse Brain

**Figure 6:**
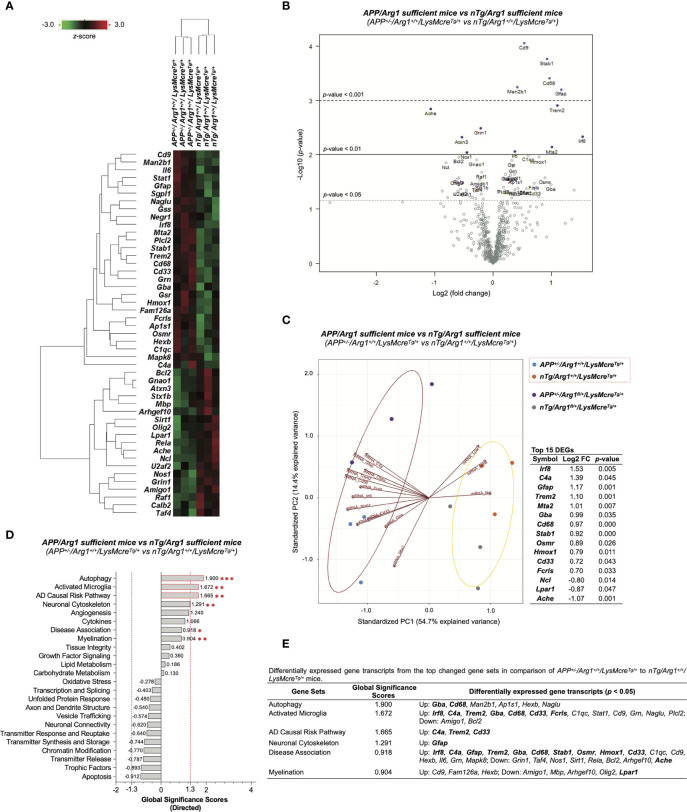
Differential gene expression analysis of overexpressing human *APP KM670/671NL* Swedish mutation in mouse brain. **(A)** A heat map with dendrogram trees represents clustering of samples and differentially expressed gene transcripts (DEGs, *p <* 0.05) by comparing *APP^+/-^/Arg1^+/+^/LysMcre^Tg/+^* to *nTg/Arg1^+/+^/LysMcre^Tg/+^* mice. The heat map uses the average Euclidean distance for the linkage and is centered and scaled by the *z*-score transformation. Red and green colors denote up and down expressed gene transcript, respectively. **(B)** A volcano plot displays all expressed genes above the background. Gene transcripts with high statistical significance stay on the top and high fold-change stay on either side. The left and right side of the volcano plot displays the down and up expressed genes, respectively. Horizontal lines indicate different thresholds of the *p*-values. The top 40 DEGs (based on *p*-values) are labeled. **(C)** A biplot of principal component analysis and a table are created by the top 15 DEGs with the highest fold-change variance. **(D)** A bar graph of gene set analysis ranks all signature pathways with directed global significance scores (GSS). The up or down regulated pathway is indicated by positive or negative GSS values. The red or blue dashed line highlights the top changed pathways based on the cut-off criterion (absolute GSS value at 1.3). The statistically changed pathways from pair-wise comparison in pathway scoring analysis are annotated with red asterisk sign (*) for up-regulation. **(E)** A table lists DEGs of the top changed pathways from both gene set analysis and pathway scoring analysis. The top 15 DEGs are bolded. Up/Down denotes up/down-regulation. n=3 samples per group representing 6 mice. The asterisk sign (*) indicates the focused pair-wise comparison of *APP* transgene genotype. *p < 0.05, **p < 0.01; ***p < 0.001. Two-way ANOVA of 2x2 factorial analysis followed by pairwise comparisons using Fisher’s PLSD. See also **Table S1**.

To measure the effect of human *APP* (*KM670/671NL*, Swedish) overexpression in *Arg1* sufficient mice, we performed differential gene expression analysis by comparing *APP/Arg1* sufficient mice to *nTg/Arg1* sufficient mice and thus identified 47 differentially expressed gene transcripts (DEGs, *p* < 0.05) (**Table S1**). A clustering heat map segregated the two groups based on these DEGs (**Figure 6A**). The top DEGs with high statistical significance and fold change variance could be visualized in the volcano plot (**Figure 6B**). Furthermore, we created PCA biplots using the top 15 DEGs based on log2 fold change. The up-regulated DEGs (*Irf8, C4a, Gfap, Trem2, Mta2, Gba, Cd68, Stab1, Osmr, Hmox1, Cd33, Fcrls*) and down-regulated DEGs (*Ncl, Lpar1, Ache*) successfully separated the two groups by a leading PC1 (54.7%) and a PC2 (14.4%) (**Figure 6C**). The *APP/Arg1* sufficient mice had increased transcriptomic pathway scores than *nTg/Arg1* sufficient mice in autophagy (*p =* 0.001, **Figure 2F**), activated microglia (*p =* 0.010, **Figure 2H**), AD causal risk pathway (*p =* 0.002, **Figure 2C**), neuronal cytoskeleton (*p =* 0.006, **Figure 2E**), disease association (*p =* 0.041, **Figure 2D**), and myelination (*p =* 0.003, **Figure 2L**) (**Figure 6D**). The DEGs of these pathways were also listed (**Figure 6E**). The GSA showed that the top 3 up-regulated pathways that met the cut-off GSS criterion were autophagy (GSS = 1.900), activated microglia (GSS = 1.672), and AD causal risk pathway (GSS = 1.665) (**Figure 6D**). We observed 13 out of the top 15 DEGs contributed to the top changed pathways identified by PSA and GSA (**Figure 6E**). Importantly, the AD causal risk pathway has 60% microglia related genes in its gene set (*Cd33, Psen2, Psmb9, Apoe, Trem2, Psmb8, Spi1, Clu, C4a*) and its up-regulated DEGs (*C4a, Trem2, Cd33*) overlapped with activated microglia pathway (**Figure 6E**). Notably, CPA showed that the *APP/Arg1* sufficient mice activated microglia/macrophages cell-type-specific gene transcripts than *nTg/Arg1* sufficient mice (**Figure 4D**). These transcriptomics data suggest that Aβ plaque activated brain myeloid cells with increased autophagy.

**Figure 7:**
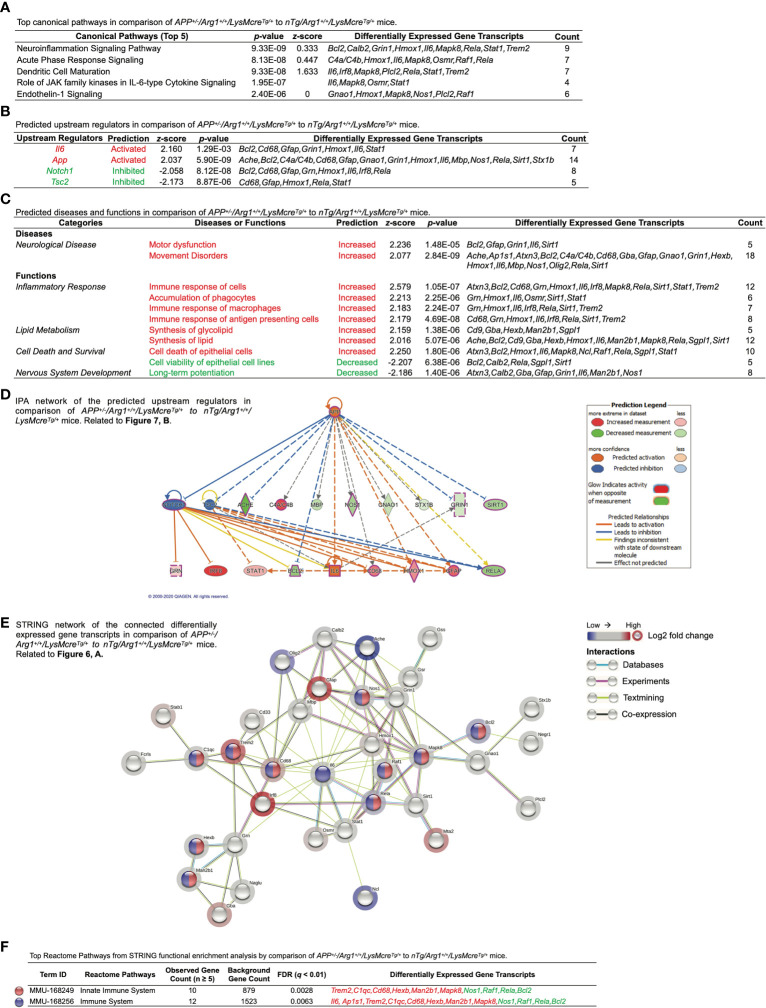
Ingenuity Pathway Analysis and STRING network analysis of overexpressing human *APP KM670/671NL* Swedish mutation in mouse brain. Differentially expressed gene transcripts (DEGs, *p <* 0.05) created by comparing *APP^+/-^/Arg1^+/+^/LysMcre^Tg/+^* to *nTg/Arg1^+/+^/LysMcre^Tg/+^* mice were analyzed by Ingenuity Pathway Analysis (IPA) and STRING network analysis using publicly available databases. **(A)** A table lists the top five canonical pathways (based on *p*-values). **(B)** A table lists the top predicted upstream regulators (based on *z*-scores). **(C)** A table lists the top predicted diseases and functions under different categories (based on *z*-scores). An absolute *z*-score value at 2.0 was set as the cut-off criterion to predict the activated/increased (*z*-score > 0) or inhibited/decreased (*z*-score < 0) states, and were highlighted in red or green. **(D)** IPA network displays the predicted upstream regulators in a hierarchical order. Upstream regulators predicted as activation or inhibition are colored in orange or blue. DEGs are colored in red or green, indicating increased or decreased expression. DEGs involved in Alzheimer’s disease are also highlighted with a purple border. Direct or indirect gene interactions are in solid or dash lines with orange or blue colors indicating activation or inhibition. The intensity of color shading represents either measured fold-change magnitude or predicted activation/inhibition magnitude. **(E)** STRING network of the connected DEGs is presented. Each node represents a DEG with a halo color of red to blue, indicating high to low values of log2 fold-change. Nodes in different colors are annotated based on the top Reactome Pathways. Each edge line represents an interaction between two DEGs based on parameters of databases (cyan), experiments (purple), textmining (yellow), and co-expression (black). **(F)** A table lists the top Reactome Pathways from STRING functional enrichment analysis. The false discovery rate (FDR) *q*-value is set at ≤ 0.01. The observed gene count is set at ≥ 5. DEGs highlighted in red or green indicate up or down expression. n=3 samples per group representing 6 mice.

Next, we performed Ingenuity Pathway Analysis (IPA) based on the DEGs mentioned above. First, IPA identified the top five canonical pathways (based on *p*-values) were neuroinflammation signaling pathway, acute phase response signaling, dendritic cell maturation, role of Jak family kinases in Il-6-type cytokine signaling, and endothelin-1 signaling (**Figure 7A**). Importantly, the neuroinflammation signaling pathway was the top activated pathway with the highest statistical significance (*p* = 9.33E-09) and the most target gene transcripts (*Bcl2, Calb2, Grin1, Hmox1, Il6, Mapk8, Rela, Stat1, Trem2*) (**Figure 7A**). Secondly, IPA predicted activated upstream regulators (*Il6, App*; *z* ≥ 2.0) and inhibited upstream regulators (*Notch1, Tsc2; z* ≤ -2.0) (**Figure 7B**). By displaying the predicted upstream regulators in a hierarchical gene interaction network, we observed that *App* was the top-notch upstream regulator that causes the entire network with the highest significance (*p* = 5.90E-09, *z* = 2.037) and the most target gene transcripts (*Ache, Bcl2, C4a/C4b, Cd68, Gfap, Gnao1, Grin1, Hmox1, Il6, Mbp, Nos1, Rela, Sirt1, Stx1b*) (**Figures 7B, D**). Moreover, IPA built two networks of regulatory effects based on the four predicted upstream regulators. One network was composed of *Il6, Notch1*, and *Tsc2*, pointing to one biological function in macrophages’ immune response with four target gene transcripts (*Grn, Hmox1, Irf8, Rela*). Another network was by *App* alone linked to one disease in motor dysfunction with five target gene transcripts (*Bcl2, Gfap, Grin1, Il6, Sirt1*). Next, we used IPA to study the top diseases/disorders and biological functions. It predicted neurological diseases with increased motor dysfunction and movement disorders (*z* ≥ 2.0) (**Figure 7C**). It also predicted that the inflammatory response was the top (based on *z*-score) increased biological function, including the increased immune response of cells, accumulation of phagocytes, immune response of macrophages, and immune response of antigen presenting cells (*z* ≥ 2.0) (**Figure 7C**). It also predicted other functions in lipid metabolism (increased synthesis of glycolipid and synthesis of lipid, *z* ≥ 2.0), cell death and survival (increased cell death of epithelial cells, *z* ≥ 2.0; decreased cell viability of epithelial cell lines*, z* ≤ -2.0), as well as nervous system development (decreased long-term potentiation, *z* ≤ -2.0) (**Figure 7C**). Lastly, we performed STRING analysis and plotted the connected DEGs to generate a gene interaction network (PPI *p*-value < 1.0E-16), highlighted by significant pathways identified by Reactome Pathways. These were innate immune system (*q* = 0.0028) and immune system (*q* = 0.0063) (**Figures 7E, F**). Overall, these data strongly suggest overexpression of human *APP* (*KM670/671NL*, Swedish) in mouse brain activates gene signatures of innate immune response associated with brain myeloid cells.

### Myeloid *Arg1* Deficiency Promotes Gene Signatures Associated With Lipid Metabolism, Myelination and Migration of Myeloid Cells in Mouse Brain During Amyloidosis

**Figure 8:**
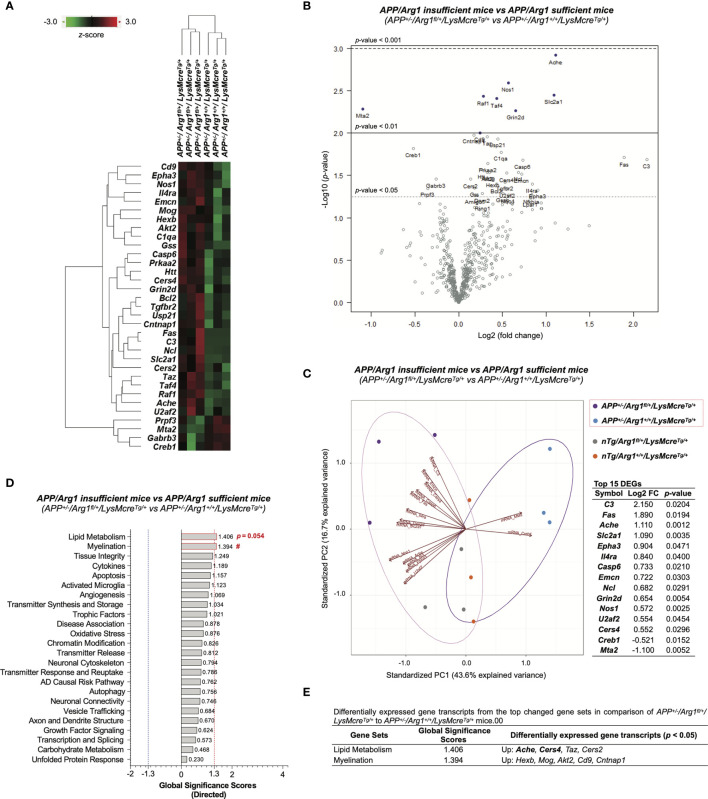
Differential gene expression analysis of *LysMcre* dependent *Arg1* haploinsufficiency in mouse brain with amyloidosis. **(A)** A heat map with dendrogram trees represents agglomerative clustering of samples and differentially expressed gene transcripts (DEGs, *p <* 0.05) by comparing *APP^+/-^/Arg1^fl/+^/LysMcre^Tg/+^* to *APP^+/-^/Arg1^+/+^/LysMcre^Tg/+^* mice. The heat map uses the average Euclidean distance for the linkage and is centered and scaled by the *z*-score transformation. Red and green colors denote up and down expressed gene transcript, respectively. **(B)** A volcano plot displays all expressed genes above the background. Gene transcripts with high statistical significance stay on the top and high fold-change stay on either side. The left and right side of the volcano plot displays the down and up expressed genes, respectively. Horizontal lines indicate different thresholds of the *p*-values. The top 40 DEGs (based on *p*-values) are labeled. **(C)** A biplot of principal component analysis and a table are created by the top 15 DEGs with the highest fold-change variance. **(D)** A bar graph of gene set analysis ranks all signature pathways with directed global significance scores (GSS). The up or down regulated pathway is indicated by positive or negative GSS values. The red or blue dashed line highlights the top changed pathways based on the cut-off criterion (absolute GSS value at 1.3). The statistically changed pathways from pair-wise comparison in pathway scoring analysis are annotated with *p*-value or red number sign (#) for up-regulation. **(E)** A table lists DEGs of the top changed pathways in gene set analysis. The top 15 DEGs are bolded. Up/Down denotes up/down-regulation. n=3 samples per group representing 6 mice. The number sign (#) indicates the focused pair-wise comparison of *Arg1* haploinsufficiency genotype. ^#^ *p* < 0.05. Two-way ANOVA of 2x2 factorial analysis followed by pairwise comparisons using Fisher’s PLSD. See also **Table S2**.

To determine the impact of reducing *Arg1* in a mouse model of amyloidosis, we obtained 33 DEGs (*p* < 0.05) in comparison of *APP/Arg1* insufficient mice to *APP/Arg1* sufficient mice (**Table S2**). The heat map of DEGs clustered the two groups separately (**Figure 8A**). The volcano plot displays the top DEGs with a high magnitude of significance and variance (**Figure 8B**). We also analyzed the PCA biplots using the top 15 DEGs (based on log2 fold change). The up-regulated DEGs (*C3, Fas, Ache, Slc2a1, Epha3, Il4ra, Casp6, Emcn, Ncl, Grin2d, Nos1, U2af2, Cers4*) and down-regulated DEGs (*Creb1, Mta2*) separated the *APP/Arg1* insufficient group from the *APP/Arg1* sufficient group with a PC1 (43.6%) and a PC2 (16.7%) (**Figure 8C**). The PSA and GSA simultaneously showed that *APP/Arg1* insufficient mice activated transcriptomic pathways in lipid metabolism (*p =* 0.054, **Figure 2G**; GSS = 1.406, **Figures 8D, E**) and myelination (*p =* 0.023, **Figure 2L**; GSS = 1.394, **Figures 8D, E**) compared to *APP/Arg1* sufficient mice.

**Figure 9:**
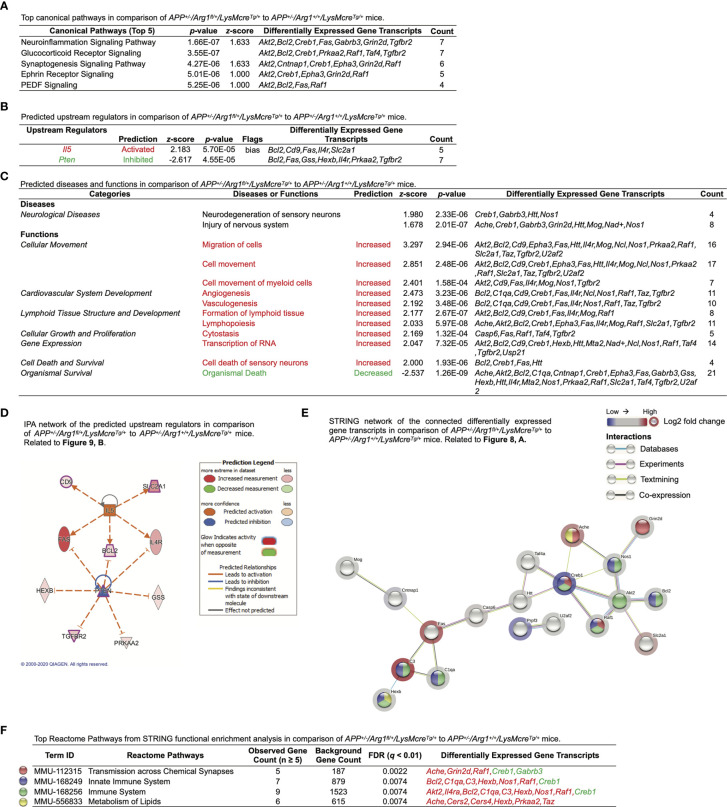
Ingenuity Pathway Analysis and STRING network analysis of *LysMcre* dependent *Arg1* haploinsufficiency in mouse brain with amyloidosis. Differentially expressed gene transcripts (DEGs, *p <* 0.05) created by comparing *APP^+/-^/Arg1^fl/+^/LysMcre^Tg/+^* to *APP^+/-^/Arg1^+/+^/LysMcre^Tg/+^* mice were analyzed by Ingenuity Pathway Analysis (IPA) and STRING network analysis using publicly available databases. **(A)** A table lists the top five canonical pathways (based on *p*-values). **(B)** A table lists the top predicted upstream regulators (based on *z*-scores). **(C)** A table lists the top predicted diseases and functions under different categories (based on *z*-scores). An absolute *z*-score value at 2.0 was set as the cut-off criterion to predict the activated/increased (*z*-score > 0) or inhibited/decreased (*z*-score < 0) states and were highlighted in red or green. **(D)** IPA network displays the predicted upstream regulators in a hierarchical order. Upstream regulators predicted as activation or inhibition are colored in orange or blue. DEGs are colored in red or green, indicating increased or decreased expression. DEGs involved in Alzheimer’s disease are also highlighted with a purple border. Direct or indirect gene interactions are in solid or dash lines with orange or blue colors indicating activation or inhibition. The intensity of color shading represents either measured fold-change magnitude or predicted activation/inhibition magnitude. **(E)** STRING network of the connected DEGs is presented. Each node represents a DEG with a halo color of red to blue, indicating high to low values of log2 fold-change. Nodes in different colors are annotated based on the top Reactome Pathways. Each edge line represents an interaction between two DEG transcripts based on parameters of databases (cyan), experiments (purple), textmining (yellow), and co-expression (black). **(F)** A table lists the top Reactome Pathways from STRING functional enrichment analysis. The false discovery rate (FDR) *q*-value is set at ≤ 0.01. The observed gene count is set at ≥ 5. Gene transcripts highlighted in red or green indicate up or down expression. n=3 samples per group representing 6 mice.

We then applied IPA on the DEGs and observed the top five canonical pathways (based on *p*-values) consisting of the neuroinflammation signaling pathway, glucocorticoid receptor signaling, synaptogenesis signaling pathway, Ephrin receptor signaling, and PEDF signaling (**Figure 9A**). Notably, the most significant neuroinflammation signaling pathway had the highest activation *z*-score ((*p* = 1.66E-07, *z* = 1.633) and the most target gene transcripts (*Akt2, Bcl2, Creb1, Fas, Gabrb3, Grin2d, Tgfbr2*) (**Figure 9A**). IPA predicted one activated upstream regulator (*Il5*, *z* ≥ 2.0, biased) and one inhibited upstream regulator (*Pten*, *z* ≤ -2.0) with an interactive gene regulation network (**Figures 9B, D**). Furthermore, IPA further built one upstream regulatory network based on *Il5* with five target gene transcripts (*Bcl2, Cd9, Fas, Il4r, Slc2a1*) and was associated with three biological functions (formation of lymphoid tissue, migration of cells, vasculogenesis). Furthermore, although no disease was predicted by IPA (*z* ≥ 2.0 or *z* ≤ -2.0), we found that neurological diseases were increased with a trend in neurodegeneration of sensory neurons (*z* = 1.980) and injury of nervous system (*z* = 1.678) (**Figure 9C**). IPA successfully predicted that the top changed function was a cellular movement in biological functions, with increased migration of cells, cell movement, and cell movement of myeloid cells (*z* ≥ 2.0) (**Figure 9C**). Other predicted functions included cardiovascular system development (increased angiogenesis and vasculogenesis, *z* ≥ 2.0), lymphoid tissue structure and development (increased formation of lymphoid tissue and lymphopoiesis, *z* ≥ 2.0), cellular growth and proliferation (increased cytostasis, *z* ≥ 2.0), gene expression (increased transcription of RNA, *z* ≥ 2.0), cell death and survival (increased cell death of sensory neurons, *z* ≥ 2.0), and organismal survival (decreased organismal death, *z* ≤ -2.0) (**Figure 9C**). Finally, the STRING network of interacted DEGs was established (PPI *p*-value = 9.28E-05) (**Figure 9E**). The STRING functional enrichment analysis identified significant pathways *via* Reactome Pathways in transmission across chemical synapses (*q* = 0.0022), innate immune system (*q* = 0.0074), immune system (*q* = 0.0074), and metabolism of lipids (*q* = 0.0074), all of which were annotated in the network (**Figures 9E, F**). Collectively, these transcriptomic findings suggest myeloid Arg1 deficiency activates gene signatures of lipid metabolism and myelination and promotes myeloid cell migration in the mouse brain of amyloidosis.

## Discussion

Together with our previous findings reporting that myeloid *Arg1* insufficiency precipitates Aβ deposition (39), the current transcriptomic analysis shows that myeloid *Arg1* insufficiency activates Aβ plaque-associated glial gene signatures to exacerbate neurodegeneration. First, we demonstrated that the *APP* transgene up-regulated pathways most related to autophagy, activated microglia, and AD causal risk, while *Arg1* haploinsufficiency up-regulated pathways of lipid metabolism and myelination. Second, we demonstrated that *APP* transgene mostly activated microglia/macrophages and myeloid Arg1 deficiency during amyloidosis promoted oligodendrocytes by analyzing cell-type-specific gene expression. Next, we provided strong evidence from analyzing key Aβ plaque-associated glial transcriptomic signatures to support the notion that *APP* transgene activated these signatures mostly by inducing disease-associated microglial genes, whereas myeloid *Arg1* haploinsufficiency increased them further by largely eliciting homeostatic microglial genes. Collectively, this is the first report to suggest that *Arg1* deficient brain myeloid cells align transcriptome signatures that may phagocytose less Aβ plaques, thus aggravating the accumulation of Aβ plaques and possibly neurodegeneration.

Microglia perform various functions, maintaining CNS homeostasis during normal aging and front-line responders and inducers for neurodegenerative diseases like AD (90, 93, 94). Earlier work from Elly Hol and others identified several Aβ associated microglial pro-inflammatory transcriptional profiles in different amyloidosis mouse models based on extensive microarray data (95–98). Eventually, all of these microglial profiles pointed to a common chronic primed microglia transcriptional signature established by gene co-expression meta-analysis (77), from which the results were built into the NanoString nCounter^®^ mouse neuropathology panel. Due to recent developments in scRNA-seq and targeted NanoString nCounter techniques, researchers have confirmed these earlier findings by further deciphering the various transcriptional subtypes and distinct stages of microglia that were interacting with Aβ plaques (56–59, 99, 100) (**Figure 5A**), thus converging age, sex, and AD risk genes as the major risk factors for AD (101, 102). Importantly, critical microglial activation genes identified in amyloidosis mice were also recently validated in human AD brains (103). Albeit with the conflicting data interpretation, microglia may show either beneficial or detrimental effects depending on aging and disease progression by inducing disease-associated microglial signatures or restoring homeostatic microglial signatures (104, 105). Microglial fitness required to dynamically switch between these states is critical for disease pathology. It was shown that the homeostatic microglial signature (HM/M0) is mostly non-phagocytic and presumably becomes suppressed to initiate the disease-associated microglial signature (DAM/MGnD) that is more phagocytic (57, 58). Therefore, microglia locked into a homeostatic state may be just as detrimental as the disease-induced state.

Our study focused on seven critical glial transcriptomic signatures involved in amyloidosis from the literature covering both homeostatic and disease-associated microglia. First, our results showed the *APP* transgene activated Aβ plaque induced genes (PIGs) as the top changed signature (**Figure 5E**) and myeloid *Arg1* deficiency during amyloidosis up-regulated it further (**Figure 5F**). The findings were in line with our previous report that myeloid *Arg1* insufficiency promoted amyloidosis (39), confirming that the magnitude of PIGs positively correlated to the load of Aβ deposition (56). Next, further investigation of individual glial signatures revealed that myeloid *Arg1* deficiency during amyloidosis preferentially activated homeostatic microglia (HM) gene signature. Therefore, we predict that the transcriptomic change in migration of myeloid cells caused by myeloid *Arg1* deficiency may be a compensatory response to the increased Aβ burden due to the non-phagocytic homeostatic microglial signature.

Recent studies found that the pre-classical M2 marker gene *Arg1* played an essential role in activities of phagocytosis and efferocytosis by myeloid cells. One study showed that *Arg1*, orchestrated by STAT6/Arg1 signaling axis, was responsible for efferocytosis of microglia/macrophages to remove dead/dying neurons (106). Another group also showed that the *Arg1* was involved in the continual efferocytosis process of engulfing and degrading apoptotic cells to provide nutrients (107). One study reported that *Arg1* was the second most up-regulated gene transcript for inducing phagocytic microglial signature (MG-dNF) relative to the non-phagocytic microglial signature (MG-nF) after injection of apoptotic neurons in mouse brain (58). Therefore, the simplicity of viewing microglial activation phenotypes as M1-like (pro-inflammatory) and M2-like (anti-inflammatory) was further challenged and has been replaced by recent scRNA-seq based discoveries of molecular subtyping microglial phenotypes (90, 104, 108, 109). These findings are consistent with our previous *in vitro* work demonstrating that repressing *Arg1* in microglial cells impaired phagocytosis (39). Thus, *Arg1* is a key microglial functional marker for facilitating phagocytosis and efferocytosis and, when suppressed, could promote non-phagocytic and homeostatic microglial signatures.

Recent progress in studying AD pathophysiology using single-cell and spatial transcriptomics in AD patients and *APP* knock-in mouse models discovered myelination-related pathways were disturbed mostly in oligodendrocytes but also in other principal CNS cells (56, 110). Our data linked myeloid *Arg1* deficiency with the myelination gene set during the amyloid challenge. First, pathway analyses revealed that myelination gene signature was up-regulated due to myeloid *Arg1* haploinsufficiency in *APP* mice (**Figures 2L** and **8D**). Second, imputing gene expression changes to CNS cells showed oligodendrocytes as one of the two significantly changed cell types. Oligodendrocyte-specific gene transcripts were only increased when the *APP* transgene was expressed in the myeloid *Arg1* haploinsufficient background mice (**Figure 4E**). Third, pathway analysis of amyloid-β associated glial transcriptomic signatures showed that the signature of Aβ plaque correlated oligodendrocyte genes (OLIGs) was up-regulated when suppressing myeloid *Arg1* in *APP* mice (**Figure 5F**). The OLIGs signature was previously reported to correlate positively with the Aβ burden in specific brain regions (entorhinal cortex and hippocampus) (56), thus suggesting that increased myelination could be a secondary effect from myeloid *Arg1* deficient mice since we previously reported these mice presented more Aβ deposition (39). Therefore, these data might suggest a potential role of *Arg1* in the crosstalk between microglia and oligodendrocytes. During conditions of neurodegenerative diseases, oligodendrocytes continue an active demyelination/remyelination process, in which microglia and reactive astrocytes both play a role (111). Our study remains unclear if/how *Arg1* deficient microglia regulate myelination during amyloidosis based on transcriptomic evidence. However, previous research demonstrated that increased *Arg1* served as a dominant switch in microglia for initiating the remyelination process (112) and that microglia could acquire a pro-regenerative state with increased *Arg1* (113, 114). Furthermore, we also found that the oligodendrocyte-specific gene transcript marker *Mog*, which was elevated in *Arg1* deficient *APP* mice in this study (**Figure 8E**), was previously identified as a critical CNS-specific autoantigen responsible for demyelination in multiple sclerosis (115, 116). Therefore, our data suggest that reduced *Arg1* in myeloid cells promotes transcript signatures associated with demyelination or delayed remyelination during the amyloid challenge.

Furthermore, our pathway analyses showed that myeloid *Arg1* haploinsufficiency during amyloidosis also up-regulated the lipid metabolism gene set (**Figures 2G** and **8D**). Lipid pathology has been validated as a shared feature between neurodegenerative mouse models and human AD (63, 117, 118). A recent study in aging and AD identified a substantial diseased microglial population termed “lipid-droplet-accumulating microglia (LDAM)”, which were defective in phagocytosis (119). The gene set analysis showed the glial signature LDAM was up-regulated in myeloid *Arg1* deficient *APP* mice without meeting significance (data not shown). While it is feasible that activated brain lipid metabolism is partly due to LDAM, this warrants further investigation. Interestingly, acetylcholinesterase (*Ache*), a therapeutic target for AD, was one of the top three gene transcripts up-regulated when reducing myeloid *Arg1* in *APP* mice (**Figures 8C, E**). It is known that Aβ peptides increased *Ache* (120), and conversely, *Ache* promoted Aβ production (121, 122), which aligned with our current finding of increased *Ache* expression with elevated Aβ plaque-induced genes. Increased *Ache* could also decrease cholinergic transmission and contributing to cognitive impairment (123). We identified the top changed network by Reactome Pathway was transmission across chemical synapses with increased *Ache, Grin2d*, and *Raf1*, and decreased *Creb1* and *Gabrb3*, all of which could contribute to cognitive dysfunction (**Figure 9F**). These findings were in line with our previous observation that myeloid *Arg1* deficiency during amyloidosis hastened mouse behavioral impairments (39).

Therefore, by analyzing gene expression profiling in fundamental neurodegeneration pathways, we provided novel transcriptomic mechanisms to corroborate the previous observation that myeloid *Arg1* deficiency exacerbated Aβ deposition by promoting gene sets essential for myelination, lipid metabolism, and activating Aβ associated glial genes biased for homeostatic/non-phagocytic microglia. By laying a foundation for the role of *Arg1* in phagocytic myeloid cells during amyloidosis, we provided a new therapeutic target for manipulating arginine metabolism through arginase 1 to benefit human AD. Considering the beneficial role of overexpressing *Arg1* in the tau transgenic mouse model (37), a future study on overexpressing *Arg1* in a mouse model of amyloidosis should be investigated. Conversely, another study using the CVN-AD mouse model (*Nos2* null) showed that sustained elevated extracellular *Arg1* level stimulated amyloidosis and promoted hippocampal neuronal death (21). These discrepant studies implicate that temporal and spatial *Arg1* activity in different CNS cell types, animal models, aging stages, and disease progression remain critical questions for future studies.

Critically, we need to mention that transcriptomic analyses cannot prove biological cell function or phenotype changes because there are many gene regulation levels besides transcription. Future studies are needed to confirm that the gene expression changes reported herein result in changes in protein levels or and that these changes modify cellular phenotype. It is important to remember that the pathway analyses identify coordinated regulation of multiple genes associated with given cell functions. Consequently, spurious errors in expression of a single gene transcript cannot explain the results we observed linking specific pathways to amyloid or *Arg1* insufficiency.

Although the LysMcre mice have been a useful tool to mainly target myeloid cells for many years (71, 124), the specificity of cell types that LysMcre targeted to suppress *Arg1* expression has been questioned recently (125). Two characterization studies on LysMcre specificity in the mouse brain were thus reported. One group showed that the LysM promoter was almost exclusively active in neurons rather than microglia within certain brain regions (neuronal layer of the forebrain motor cortex and granule cell layer of the cerebellum), but on average, it was active in less than 30% of both neurons and microglia across the whole brain (126). Another recent report found that the LysMcre promoter was active in 40% of macrophage/microglia and only 8% of neurons in adult mouse retina (127). Although the LysMcre promoter was still validated to express greatest in myeloid cells, both studies provided evidence to show the existence of recombination in neurons, albeit to different levels.

Although the functional role of the antibacterial enzyme LysM in neurons is still unclear, recent scRNA-seq studies in microglia showed that *Lyz2* encoding LysM was one of the commonly induced microglial genes during neurodegenerative diseases (104). *Lyz2* was up-regulated in disease-associated microglial signatures such as PIGs (56), DAM (57), and MGnD (58), indicating that the brain myeloid cells most likely up-regulate *Lyz2* as a compensatory response to amyloid stimulation. These recent findings using scRNA-seq in amyloid-depositing mouse models confirmed one previous study in human AD, which showed that lysozyme protein was increased in the CSF of AD patients, co-localized with Aβ plaque in postmortem AD brains, and directly interacted with Aβ *in vitro* (128). The increase of secreted lysozyme in CSF was thus attributed to the mononuclear monocytes/macrophages. Therefore, the current evidence suggests a protective role of up-regulating lysozyme in responding to Aβ, a process that mainly occurs in brain myeloid cells rather than other CNS cells including neurons. These findings indicate that LysMcre dependent *Arg1* haploinsufficiency has a functional consequence in brain myeloid cells. However, it is possible that the one allele deletion of *Lyz2* due to the insertion of Cre-recombinase also caused unknown effects. Since all the mice shared the same *Lyz2* haploinsufficient background, the unintended expression should be present in all mice and should minimize putative effects on differentially expressed genes. In the future, mouse lines like the Cx3cr1-CreERT2 (129, 130) for monocytes/macrophages or Tmem119-CreERT2 (131) and Tmem119-tdTomato reporter (132) for resident microglia may be favored because they have not been shown to have similar caveats to date.

Another limitation to our study is that we analyzed bulk RNA samples. It is possible that subtle changes in myeloid gene expression were masked by dilution with RNA from other cell types. On the other hand, mechanical dissociation of myeloid cells has other caveats, such as the possibility that disease-associated microglia or microglia adjacent to amyloid deposits are more fragile and difficult to isolate. This study intended to understand how myeloid *Arg1* insufficiency impacted overall CNS gene expression in neurodegeneration. Future studies should increase replication with a larger sample size and compare these targeted transcriptome results with larger transcriptome dataset to ensure the effects of myeloid *Arg1* deficiency during amyloidosis.

Overall, our findings suggest that myeloid *Arg1* haploinsufficiency elevates Aβ associated genes enriched in brain myeloid cells and oligodendrocytes. Deficiency of *Arg1* in brain myeloid cells preferentially promotes a transcriptomic signature that is more homeostatic and less phagocytic, possibly inhibiting their crucial transition from a homeostatic to a disease-associated state during amyloidosis, leading to more Aβ deposition. Future therapeutics to modulate arginase 1 in brain myeloid cells may provide potential disease-modifying treatment for AD patients.

## Data Availability Statement

The datasets generated for this study are included in the supplemental documents and are also available from the corresponding author upon request. The raw nCounter data are publicly available through the National Center for Biotechnology Information (NCBI) Gene Expression Omnibus (GEO) under accession number GSE172108.

## Ethics Statement

The animal study was reviewed and approved by Institutional Animal Care and Use Committee (IACUC) in the University of South Florida and the University of Kentucky.

## Author Contributions

CM contributed to the design and implementation of the research, performed bioinformatic analyses with nSolver, IPA and STRING, statistical analysis using SPSS, interpreted data, and wrote the first draft of the manuscript. JH contributed to the breeding of mice, harvesting mouse brains, and statistical analysis using SPSS. AK, HL, and M-LS contributed to the mRNA extraction and preparation. MG contributed to the breeding and genotyping of the mice. MO, BZ, JG, and DF contributed to the characterization of mouse lines. MG, DM, PB, and DL contributed to the design, conceptualization of the research, interpretation of the data, and writing of the manuscript. All authors contributed to the article and approved the submitted version.

## Funding

Funding for this work was provided by the NIH R21-AG055996 (to DL), R01-AG054559 (to DL), R01-AG051500 (to DM), R01-NS091582 (to JG), R01-AI095307 (to DF), Alzheimer’s Association AARGD-16-441534 (to DL), and MNIRGD-12-242665 (to DL), Florida Department of Health Ed and Ethel Moore Alzheimer’s disease (8AZ30) (to DL and PB), and IKBX004214 (to PB). CM was awarded by USF Health Neuroscience Institute Dorothy Benjamin Graduate Fellowship in Alzheimer’s Disease.

## Conflict of Interest

The authors declare that the research was conducted in the absence of any commercial or financial relationships that could be construed as a potential conflict of interest.

## Supplementary Material

Supplementary Figure 1 Overview of all normalized data. All data were normalized for gene transcript expression against *APP* transgene genotype and *Arg1* haploinsufficiency genotype. **(A)** A heat map of all normalized data *via* unsupervised clustering of mouse genotypes and a condensed heat map for unsupervised clustering of 770 genes. All data passed QC metrics without any flags (top bar, yellow). Genes expressed below the background threshold are flagged in blue. The orange or blue color in the heat map indicates high or low gene expression *z*-score of each sample. All scores are presented on the same scale *via* a *z*-transformation. **(B)** Principal components of all normalized data. The principal component analysis shows the four groups’ variance using the four leading components based on all normalized data. **(C)** A heat map of correlation matrix of all signature pathways from pathway scoring analysis was presented. Yellow and blue colors indicate positive and negative correlation and thus aggregate separately. Pathways that show statistically significant main *APP* transgene genotype effects are highlighted in red or blue text to indicate up or down-regulation, respectively.

## Abbreviations

PSA, Pathway Scoring Analysis; GSA, Gene Set Analysis; CPA, Cell-type Profiling Analysis.
