# Supplementary figures and images for: Myeloid Arginase 1 Insufficiency Exacerbates Amyloid-β Associated Neurodegenerative Pathways and Glial Signatures in a Mouse Model of Alzheimer’s Disease: A Targeted Transcriptome Analysis
Source: Front Immunol. 2021 May 11;12:628156. doi: 10.3389/fimmu.2021.628156 (PMC8144303; doi:10.3389/fimmu.2021.628156)

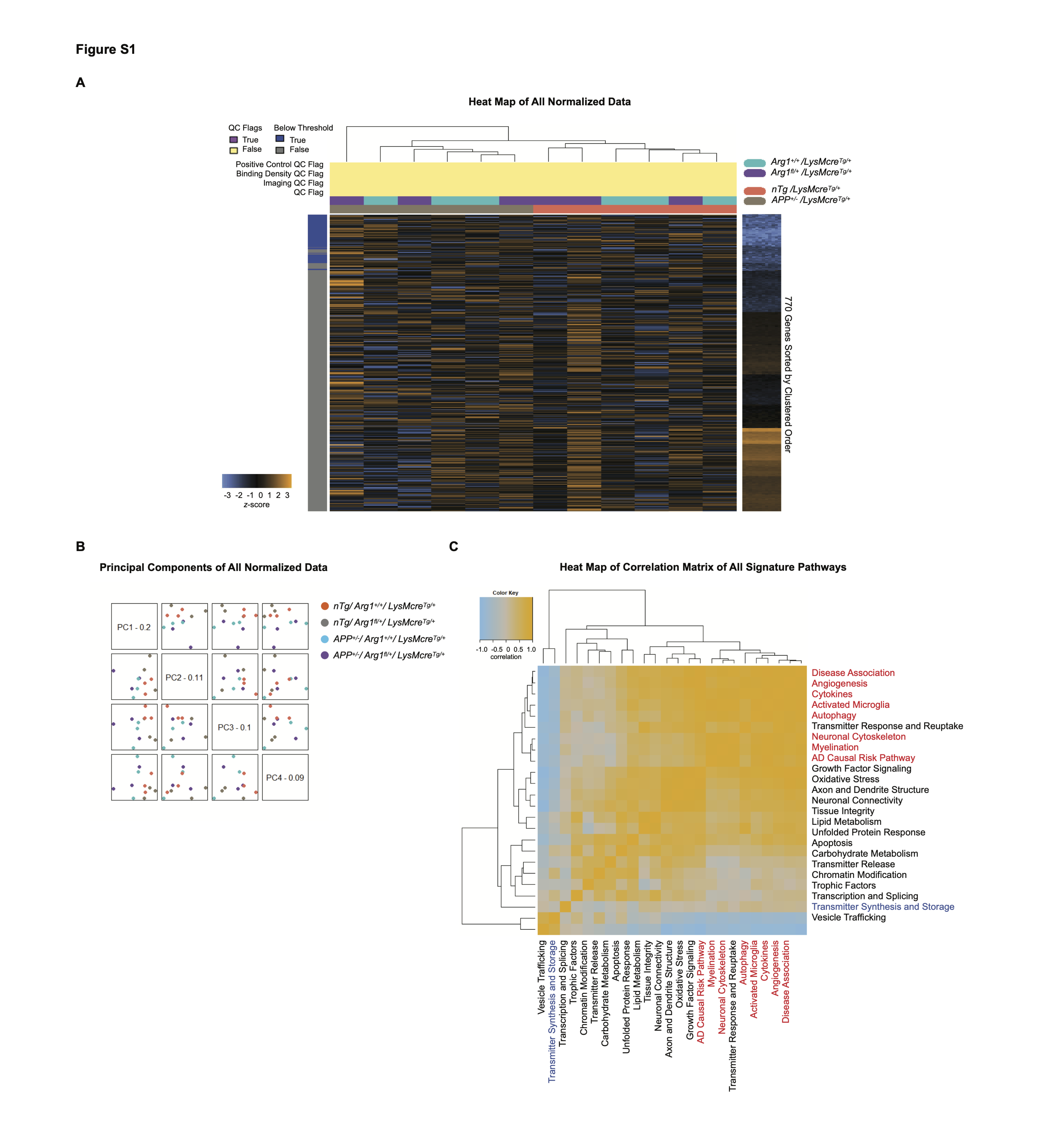

Supplement: Supplementary Figure 1 — Overview of all normalized data. All data were normalized for gene transcript expression against APP transgene genotype and Arg1 haploinsufficiency genotype. (A) A heat map of all normalized data via unsupervised clustering of mouse genotypes and a condensed heat map for unsupervised clustering of 770 genes. All data passed QC metrics without any flags (top bar, yellow). Genes expressed below the background threshold are flagged in blue. The orange or blue color in the heat map indicates high or low gene expression z-score of each sample. All scores are presented on the same scale via a z-transformation. (B) Principal components of all normalized data. The principal component analysis shows the four groups’ variance using the four leading components based on all normalized data. (C) A heat map of correlation matrix of all signature pathways from pathway scoring analysis was presented. Yellow and blue colors indicate positive and negative correlation and thus aggregate separately. Pathways that show statistically significant main APP transgene genotype effects are highlighted in red or blue text to indicate up or down-regulation, respectively. [file Image_1.tiff]
